# A brief video intervention targeting self-stigma among childhood maltreatment survivors: an international, multicentre, single-blind, randomised controlled trial

**DOI:** 10.1016/j.eclinm.2026.104201

**Published:** 2026-09-15

**Authors:** Shilat Haim-Nachum, Misari Oe, Soraya Seedat, Fatima Ahmed, Chana T. Fisch, Amit Lazarov, Dan Jenkins, Julia Carranza-Neira, Leslie Young, Stefanie Rita Balle, Yaron Sela, Yuval Neria, Vedat Şar, Görkem Ayas, Billy Jansson, Ulrich Schnyder, Monique Pfaltz, Doron Amsalem

**Affiliations:** a The School of Social Work, Tel-Aviv University, Tel-Aviv, Israel; b The Sagol School of Neuroscience, Tel Aviv University, Tel-Aviv, Israel; c Department of Psychiatry, New York State Psychiatric Institute, Columbia University Irving Medical Center, New York, NY, USA; d Department of Neuropsychiatry, Kurume University School of Medicine, Japan; e Stellenbosch University/South African Medical Research Council Unit on the Genomics of Brain Disorders, Department of Psychiatry, Faculty of Medicine and Health Sciences, Stellenbosch University, Cape Town, South Africa; f School of Psychological Sciences, Tel Aviv University, Tel-Aviv, Israel; g Department of Psychology, University of Fribourg, Fribourg, Switzerland; h Faculty of Medicine, Universidad de Piura, Lima, Peru; i Department of Psychology, LMU Munich, Germany; j Department of Health Promotion, School of Public Health, Faculty of Medical and Health Sciences, Tel Aviv University, Tel Aviv, Israel; k Department of Psychiatry, School of Medicine, Koç University, Istanbul, Türkiye; l Graduate School of Health Sciences, Koc University, Istanbul, Türkiye; m Department of Psychology, Institution for Pedagogics, Psychology and Social Work, Mid Sweden University, Östersund, Sweden; n University of Zurich, Zurich, Switzerland

**Keywords:** Childhood maltreatment, Self-stigma, Brief-video intervention, Help-seeking, Multi-national study

## Abstract

**Background:**

Self-stigma is a barrier to treatment-seeking among survivors of childhood maltreatment. Although brief online interventions have effectively reduced stigma toward mental disorders, few have targeted self-stigma among childhood maltreatment survivors, with none being tested across multiple countries. Here we examined whether a brief, social contact-based video could reduce self-stigma and increase help-seeking intentions across six countries.

**Methods:**

In this completed, international, multicentre, single-blind, randomised controlled trial (ClinicalTrials.gov: NCT06159075) with 1:1 allocation and concealed assignment, young adults with a history of childhood maltreatment from the USA, Sweden, Switzerland, Japan, South Africa, and Türkiye were randomly assigned to either a social-contact video intervention (featuring a survivor's personal story acknowledging shame and modelling recovery) or a length-matched psychoeducation control. Participant blinding was not feasible; participants were unaware of the alternative condition, and investigators and analysts were blinded throughout. Prespecified primary outcomes were self-stigma (comprising of Alienation, Stereotype Endorsement, Secrecy, Perceived Recovery) and help-seeking intentions, assessed before and after intervention and at 30-day follow-up; following peer review, Perceived Recovery was designated the focal outcome for hypothesis testing, with the remaining domains analysed as secondary outcomes. Between-group differences at each timepoint were estimated using linear mixed-effects models adjusting for baseline scores. Adverse events were not assessed; no harms were reported.

**Findings:**

Enrolment began on April 1st, 2024, and was completed on August 29, 2025. The intention-to-treat analysis included 2499 participants (mean age 27.1 years, *SD* 5.1; 1488 (61.9%) female). The social contact intervention produced a small but statistically significant improvement in Perceived Recovery relative to the psychoeducational control after intervention (adjusted mean difference [AMD] 0.201, 95% CI 0.076–0.327; p = 0.0020). No significant between-group differences emerged for Alienation, Stereotype Endorsement, Secrecy, or Help-Seeking Intentions, and all remained non-significant after Benjamini–Hochberg false discovery rate correction. Among US participants—the only site with sufficient follow-up data—no significant between-group difference was observed in Perceived Recovery at 30-day follow-up (AMD 0.050, 95% CI −0.306 to 0.407; p = 0.78), indicating that a single brief exposure was insufficient to produce durable change.

**Interpretation:**

A single social contact-based video did not reduce most self-stigma domains or improve help-seeking relative to a psychoeducational control. More intensive or targeted approaches are needed to meaningfully address self-stigma among childhood maltreatment survivors.

**Funding:**

SHN received support from the Azrieli Foundation Early Career Faculty Fellowship. Data collection in the USA was funded from discretionary research funds at 10.13039/100014546NYSPI. Regarding data collection in Japan, this work was supported by 10.13039/501100001691JSPS, 10.13039/501100001691KAKENHI Grant Number JP 23K02998. In South Africa, the study was supported by the 10.13039/501100001322South African Medical Research Council Unit on the Genomics of Brain Disorders. Data collection costs in other countries were covered by the participating institutions/researchers.

**Research in context**

**Evidence before this study**

Evidence before this study was identified through searches of PubMed, PsycINFO, and Web of Science from October 1st 2023 through 30 January 2024, using terms combining childhood maltreatment, self-stigma, and intervention, with no language restrictions. Brief social contact–based video interventions have demonstrated effectiveness in reducing public stigma across mental health conditions, but interventions targeting self-stigma among childhood maltreatment survivors specifically are scarce, typically small, conducted in single countries, and focused predominantly on survivors of sexual or physical abuse.

**Added value of this study**

To our knowledge, this is the first large-scale, multi-country randomised controlled trial examining a brief social contact–based video intervention to reduce self-stigma among young adults with a history of childhood maltreatment. Across six culturally and socioeconomically diverse countries, a single 2-min narrative video produced a small between-group improvement in recovery-oriented self-beliefs.

**Implications of all the available evidence**

Brief, scalable, social contact–based video interventions may provide a feasible global strategy for improving recovery-related beliefs among childhood maltreatment survivors. However, their limited effects on other aspects of self-stigma and help-seeking intentions suggest that more intensive or targeted approaches are needed and that cultural context should be considered when designing stigma-reduction interventions. Future research should examine how to strengthen and sustain these effects through repeated exposure, cultural adaptation, and integration with additional support mechanisms.

## Introduction

Childhood maltreatment, including emotional, physical, and sexual abuse, as well as emotional or physical neglect, is a global public health problem. Childhood maltreatment is associated with profound and lasting effects on mental health, including depression, anxiety, posttraumatic stress disorder (PTSD), and personality disorders.^1^, ^2^, ^3^, ^4^, ^5^, ^6^, ^7^, ^8^^,^^9^ Beyond diagnosable disorders, childhood maltreatment survivors commonly report shame, hopelessness, and feelings of unworthiness or being undeserving of love^10^, ^11^, ^12^, ^13^ - cognitive and emotional sequelae that compound the barriers to recovery and further diminish quality of life.

Despite these profound effects, many childhood maltreatment survivors often avoid seeking professional help^14^ on account of both structural (e.g., cost, access to mental health services, availability) and psychological barriers (e.g., shame, guilt, distrust, self-blame). Self-stigma refers to the internalisation of negative societal stereotypes and prejudices, leading individuals to apply these beliefs to themselves in the form of shame, self-blame, and guilt.^15^ It is one of the most pivotal and persistent obstacles to help seeking, including among survivors of childhood maltreatment specifically, due to shame, anticipated rejection, and distrust of services.^15^, ^16^, ^17^ Moreover, self-stigma also impairs functioning in family and work domains^18^^,^^19^ and contributes to intergenerational transmission of childhood maltreatment.^20^ Self-stigma also correlates with poorer treatment adherence^21^ and negative clinical outcomes including depression, suicidality, and reduced quality of life.^22^^,^^23^ Taken together, these findings underscore the need for targeted interventions to reduce self-stigma and enhance openness to treatment among childhood maltreatment survivors.

One such potential targeted intervention is the social contact-based intervention [see^24^] which typically involve a member of a stigmatised group sharing a personal narrative that integrates both distress and recovery. This intervention is thought to work by evoking emotional engagement and offering a credible counterexample that moderately disconfirms entrenched beliefs (e.g., “people like me cannot recover” or “seeking help means weakness”) without triggering defensive dismissal.^25^^,^^26^ By eliciting empathic resonance while gently challenging entrenched negative self-schemas, such interventions can weaken self-stigmatising cognitions and enhance readiness for help-seeking. Social contact-based interventions, particularly those that emphasise personal storytelling and connection, have been found to be highly effective in reducing stigma and promoting openness to treatment-seeking among diverse populations, such as individuals with mental illness, substance use disorders, and military veterans with PTSD.^27^

Recent studies have demonstrated that brief (60–120 s) online social contact videos can effectively reduce public stigma towards individuals living with depression,^28^ psychosis,^29^ schizophrenia,^30^ and anxiety and depression.^31^^,^^32^ However, these interventions have primarily targeted public stigma, rather than self-stigma.^33^, ^34^, ^35^

Evidence-based digital interventions specifically designed to address self-stigma are still relatively scarce,^11^^,^^34^ with existing research also presenting two key limitations that warrant attention. First, existing research on self-stigma among childhood maltreatment survivors has largely focused on survivors of sexual and physical abuse, while experiences of neglect and emotional abuse remain comparatively understudied, particularly outside Western contexts.^36^ Second, most studies were conducted in the USA, limiting their cross-cultural/national generalisability.^37^ Indeed, cross-national data reveal large disparities in help-seeking rates and stigma mechanisms.^14^

To date, no large-scale global initiative has systematically sought to reduce self-stigma among survivors of child abuse including neglect. Aiming to address this gap, while recognising the global prevalence of childhood maltreatment and the pervasive stigma surrounding it, we assembled a consortium of leading childhood maltreatment researchers from both Western, Educated, Industrialised, Rich, and Democratic and non-WEIRD countries, including researchers from Japan, South Africa, Sweden, Switzerland, Türkiye, and the USA. A recent pilot study conducted in our lab demonstrated that a similar 2-min online social contact-based video successfully reduced self-stigma among young American adults who experienced childhood maltreatment,^11^ and video-based interventions have shown particularly large effects among young adults aged 18–35.^11^^,^^38^ The present randomised controlled trial aimed to examine whether a brief social-contact video intervention could reduce self-stigma and improve help-seeking attitudes among adults with a history of childhood maltreatment across six culturally diverse countries, compared with a psychoeducational control condition. The pre-registered primary outcomes included Self-Stigma (comprised of Perceived Recovery, Alienation, Stereotype Endorsement, Secrecy), and Help-Seeking Intentions and were assessed immediately after intervention and at 30-day follow-up. Given concerns regarding the conceptual heterogeneity and psychometric properties of the self-stigma components, analyses examined the individual self-stigma subscales rather than the composite score. We also examined whether intervention effects varied according to participant characteristics and engagement with the intervention.

## Methods

### Study design and participants

This was a two-arm, parallel-group, individually randomised controlled superiority trial conducted across six countries (USA, Sweden, Switzerland, Japan, South Africa, and Türkiye). The study was prospectively preregistered at ClinicalTrials.gov [NCT06159075] before data collection. The trial was single-blind (assessor-blinded): participants could not be blinded to the video they viewed. Recruitment and trial procedures were conducted at six university-affiliated research sites across the six participating countries. Eligibility criteria required participants to be aged 18–35 years, resident in the study country, proficient in the local language, and to self-report at least one form of childhood maltreatment. Gender was assessed via self-report, with participants asked to indicate their gender identity (e.g., male, female, or prefer not to answer); sex assigned at birth was not collected. Childhood maltreatment was defined as having experienced physical, emotional, or sexual abuse, or physical or emotional neglect before age 18, assessed using screening items derived from the Adverse Childhood Experiences (ACE) questionnaire. Participants failing more than one attention check (e.g., “Please choose ‘I agree’”) or “failing CAPTCHA verification were excluded. These criteria are consistent with those specified in the registered protocol.

### Ethics

This study was approved by the Tel Aviv University ethics committee (approval number 0009168-1), the New York State Psychiatric Institute Institutional Review Board (Protocol #8453, PI: Yuval Neria), Stellenbosch University's ethics review board (Ethical Clearance number M24/04/007), and Kurume University's Ethics Committee Concerning Medicine (reference number 23195). Sites in Switzerland (Zurich), Sweden, and Türkiye did not undergo formal ethics committee review, as the research team and their respective ethics boards determined the anonymous, non-interventional, online nature of data collection at these sites did not meet the threshold requiring such review. The study was conducted in accordance with the Declaration of Helsinki. Informed consent was obtained from all participants before their inclusion in the study.

### Randomisation and masking

In the USA, Japan, Switzerland, and Sweden, participants were recruited exclusively via Cloud Research and Amazon Mechanical Turk (MTurk), and were compensated for their efforts according to the amount agreed upon before entering the study, as determined by the standards for their respective countries. In South Africa participants were recruited through social media platforms and were similarly compensated for their efforts. Turkiye recruitment took place through snowballing via email distribution lists, academic courses, and social media platforms and compensation was not provided. While recruitment methods varied by country due to differences in the availability of online crowdsourcing platforms; all sites applied identical eligibility criteria and randomisation procedures regardless of recruitment modality. Participants were randomly assigned to either a human-narrated social-contact intervention or psychoeducational control condition using a computer-generated allocation sequence implemented by Qualtrics at the point of entry to the survey, in a 1:1 ratio, following confirmation of eligibility. Allocation was concealed from both participants and investigators through automated assignment by the platform; participants were unaware of which condition they would receive prior to randomisation. Following randomisation, blinding of participants to their assigned condition was not feasible, as participants viewed the video within the same session. All investigators and data analysts were blinded to group allocation, and the senior biostatistician was masked to study hypotheses throughout the analysis.

### Procedures

Participants assigned to both the human-narrated social-contact intervention or psychoeducational control condition viewied a single 2-min video within the same online session. No treatment-as-usual control was included, because this was a brief behavioural intervention study, rather than a clinical treatment trial. Immediately after viewing the video, participants rated their emotional engagement and completed the same self-stigma and treatment-seeking measures.

The 2-min social contact-based intervention video featured a young female actress in her twenties who shared a scripted narrative that described her difficulties coping with childhood maltreatment experiences (“*growing up in a violent house wasn't easy*”… “*I hardly ever felt safe or wanted… I mostly felt lonely and unseen*”). She expressed emotions of guilt and shame when thinking about sharing her story with others and reflected on the misconceptions she had had about seeking treatment (“*I was embarrassed to tell anyone that I was abused by the people who were supposed to protect me… I felt this strong sense of guilt and shame. I just blamed myself… I knew it would impact on how people thought about me*”). The video concluded with themes of healing and optimism, as the actress shared the benefits she experienced from attending therapy. It concluded with a supportive and encouraging message: “*I started learning how to get on with my life. There's a whole community of people in this world that share a similar story. I'm not alone anymore*.”

The social contact-based intervention was designed according to the principle of moderate disconfirmation, that is, presenting information that challenges but does not invalidate existing maladaptive beliefs. This approach, central to social-contact–based stigma reduction,^25^^,^^26^ aims to evoke emotional engagement and cognitive re-evaluation while minimising defensiveness or rejection of the message. By depicting a relatable peer who acknowledges shame and fear before modelling recovery and connection, the video aims to provide an emotionally engaging yet believable counterexample that gently disconfirms self-stigmatising beliefs (“people like me cannot recover” or “seeking help means weakness”).

The intervention was developed in accordance with established best practices for digital social contact interventions and with input from lived-experience experts as well as faculty experts in psychiatry, behavioural science, and media design.

Linguistic translation and country-specific adaptation of the two videos (intervention, control) followed a standardised three-step translation protocol: 1) Forward–backward translation by two bilingual translators; 2) Script-to-script reliability assessment by bilingual raters (Visual Analogue Scale, 0–100) evaluating equivalence to the English version (agreement ≥80); and 3) Video-to-video reliability assessment: Six raters evaluated the consistency of video delivery across languages (e.g., English vs. translated versions). The raters alternated the viewing order to prevent bias, and ≥80% agreement was necessary to ensure reliable delivery and interpretation of the content. Discrepancies prompted revision until consensus was achieved.

The psychoeducational control video was matched to the intervention in duration and delivery format. It presented narrated text slides over neutral visuals describing common psychological effects of childhood maltreatment, including shame, self-blame, and difficulties seeking help, as well as general information about self-stigma and the potential benefits of mental health care. It did not include a personal narrative, a survivor protagonist, or social-contact elements intended to evoke identification or empathy.

A timer was implemented to ensure participants could not skip the video. Assessments were conducted at baseline, immediately after the social contact intervention, and at a planned 30-day follow-up. However, follow-up data were incomplete across several countries due to logistical and technical constraints during data collection. As a result, the prespecified within-participant follow-up analyses could not be conducted for most sites and were limited to the USA, where sufficient longitudinal data were available. Consequently, the primary analyses presented here focus on pre–post-intervention changes. Due to the brief nature of the intervention and immediate post-intervention assessment, no attrition occurred between randomisation and primary outcome measurement. No adverse events or serious adverse events were formally monitored or reported by participants or site investigators during or following the study.

Self-stigma was assessed using four subscales, each adapted for childhood maltreatment experiences. *Secrecy* (3 items) measured the inclination to conceal one's childhood maltreatment history.^39^ *Stereotype Endorsement* and *Alienation* (4 items each) were adapted from the Internalised Stigma of Mental Illness scale.^40^ *Perceived Recovery* (4 items) was drawn from the Recovery Assessment Scale.^41^ All items were rated 1–4 (strongly agree to strongly disagree); higher scores indicate greater self-stigma. Internal consistency ranged from *α* = 0.42 to 0.81 across subscales (see Table 1); the low reliability of Secrecy (*α* = 0.42) warrants cautious interpretation of findings for that subscale. A total self-stigma composite was not computed. The four subscales are conceptually heterogeneous, spanning concealment behaviour (Secrecy), belief endorsement (Stereotype Endorsement), social alienation (Alienation), and recovery expectations (Perceived Recovery), and their aggregation would obscure theoretically meaningful distinctions. The low reliability of the Secrecy subscale (*α* = 0.42) further precludes its inclusion in a composite without introducing psychometric distortion. This separation is additionally supported by the confirmatory factor analysis reported below, in which the five-factor correlated model yielded acceptable fit (CFI = 0.947; TLI = 0.935; RMSEA = 0.047), indicating the constructs are empirically separable. Analysing subscales independently also preserves interpretive specificity: a composite would have diluted the domain-specific effect on Perceived Recovery, which maps directly onto the intervention's narrative arc of hopefulness and agency. Openness to seeking treatment was assessed using the Attitudes Toward Seeking Professional Psychological Help Scale—Short Form [ATSPPH-SF],^42^ a three-item scale rated1-4 (higher scores reflect greater willingness to seek treatment; *α* = 0.74). Emotional engagement with the video was assessed using the three-item Emotional Engagement Scale [EES],^43^ adapted from Amsalem et al.,^25^ rated1-4 (higher scores reflect greater emotional engagement; *α* = 0.84).

**Table 1:** Scale items, sources, and internal consistency (Cronbach's *α*).

Construct	Subscale	Item (modified for CM experiences)	Original source	Cronbach's *α*
Self-Stigma	Secrecy	1. A person who was abused during childhood should not tell other people about it.	Link et al.^38^	*α* = 0.42
		2. If you have ever been treated for abuse, the best thing to do is to keep it a secret.		
		3. In view of society's negative attitudes toward people who were abused, you would advise these people to keep it a secret.		
	Stereotype Endorsement	1. People who were abused during childhood cannot live a good, rewarding life.	Ritsher et al.^39^	*α* = 0.75
		2. People who were abused during childhood shouldn't get married.		
		3. Because I was abused as a child, I need others to make most decisions for me.		
		4. I can't contribute anything to society because I was abused as a child.		
	Alienation	1. I am embarrassed or ashamed that I was abused as a child.	Ritsher et al.^39^	*α* = 0.81
		2. I am disappointed in myself for being abused as a child.		
		3. I feel inferior to others who were not abused during childhood.		
		4. People who were not abused could not possibly understand me.		
	Perceived Recovery	1. I can handle what happens in my life	Corrigan et al.^40^	*α* = 0.73
		2. Something good will eventually happen		
		3. I'm hopeful about my future		
		4. I am willing to ask for help		
Help-Seeking Intentions		1. I might want to have psychological counselling I the future	Elhai et al.^41^	*α* = 0.74
		2. I would want to get psychological help if I were worried or upset for a long period of time		
		3. A person with an emotional problem is not likely to solve it alone, he or she is more likely to solve it with professional help		
Emotional Engagement		1. It made me happy to watch the video	De Vreede et al.^42^	*α* = 0.84
		2. I care about the contents of this video		
		3. This video had a positive impact on my mood		

A five-factor correlated Confirmatory Factor Analysis was estimated on the pre-intervention item responses. Each factor was identified by fixing its first indicator loading to 1.0. The five-factor correlated model yielded acceptable to good fit: *χ*^2^(125) = 810.28, p < 0.0001; CFI = 0.947; TLI = 0.935; RMSEA = 0.047. The CFI, TLI (≥0.90), and RMSEA (<0.05) all fall within acceptable ranges, supporting the hypothesised five-factor structure in this sample.

#### Outcomes

The prespecified primary outcomes were changes from baseline in self-stigma and Help-Seeking Intentions, assessed immediately after the intervention and at the planned 30-day follow-up. Self-stigma was assessed using four conceptually distinct domains: Perceived Recovery, reflecting hope, agency, and beliefs concerning the possibility of recovery; Alienation, reflecting feelings of social disconnection and not belonging; Stereotype Endorsement, reflecting endorsement of negative stereotypes concerning individuals who have experienced childhood maltreatment; and Secrecy, reflecting the inclination to conceal one's childhood maltreatment history. Help-Seeking Intentions reflected participants' willingness to seek professional psychological help. Following concerns regarding outcome multiplicity, Perceived Recovery was designated as the focal outcome for hypothesis testing because it most closely aligned with the intervention's recovery-oriented theoretical framework. Alienation, Stereotype Endorsement, Secrecy, and Help-Seeking Intentions were treated as secondary outcomes, with p values adjusted using the Benjamini–Hochberg false discovery rate procedure. Emotional engagement with the video was assessed immediately after the intervention as an additional exploratory outcome. The protocol also prespecified the hypothesis that intervention effects would be similar across countries; country-level analyses therefore examined this prespecified cross-cultural hypothesis.

### Statistics

Analyses were conducted using SPSS (version 29.0). Sample size was determined a priori using G∗Power 3.1, based on the effect size observed in the US pilot trial (Cohen's *d* = 0.15), *α* = 0.05 (two-tailed), and power = 0.80. For a two-sample between-group comparison, this yields a required sample of 698 per group (total 1396). The pooled randomised sample of 2499 (control n = 1,241, intervention n = 1258) substantially exceeds the minimum required for the global primary analysis, yielding an achieved power of 96.3% for Perceived Recovery at the pre-specified effect size. Some sites required screening more than 400 individuals to reach 400 eligible participants meeting childhood maltreatment history criteria. A total of 2499 participants were randomised; after exclusions for failed attention checks or CAPTCHA verification.

Intervention effects were estimated using linear mixed-effects models (LME), with baseline (pre-intervention) score entered as a fixed covariate, post-intervention score as the dependent variable, and Group as a fixed effect; the Group coefficient from this model yields the adjusted mean difference (AMD) after intervention, the between-group difference in post-intervention scores after accounting for baseline. A random intercept was included per participant. The trial was preregistered with self-stigma (assessed across four domains: Alienation, Stereotype Endorsement, Secrecy, and Perceived Recovery) and Help-Seeking Intentions as primary outcomes. Following concerns regarding outcome multiplicity raised during peer review, we reduced emphasis on omnibus interpretation and retained Perceived Recovery as a theoretically motivated focal subscale analysis because it most closely aligned with the intervention's recovery-oriented theoretical framework and reflected the intervention's hypothesised mechanism, namely promoting hope, agency, and recovery-oriented beliefs.^44^ Other self-stigma subscales (Alienation, Stereotype Endorsement, Secrecy) and Help-Seeking Intentions were treated as secondary outcomes and were corrected for multiple comparisons using the Benjamini–Hochberg false discovery rate (BH-FDR) procedure (*k* = 4; *α* = 0.05). Models were estimated using restricted maximum likelihood (REML). Statistical significance was set at p < 0.05 (two-tailed) throughout; BH-FDR adjusted p values (p) are reported alongside unadjusted values for secondary outcomes. Exploratory analyses examined emotional engagement with the video and whether intervention effects varied across countries and participant subgroups.

Analyses were conducted under an intention-to-treat (ITT) framework, including all N = 2499 randomised participants.

### Role of the funding source

The funders had no role in study design, data collection, data analysis, interpretation of findings, or writing of the report. The corresponding author had full access to all data and had final responsibility for the decision to submit for publication.

## Results

**Fig. 1:**
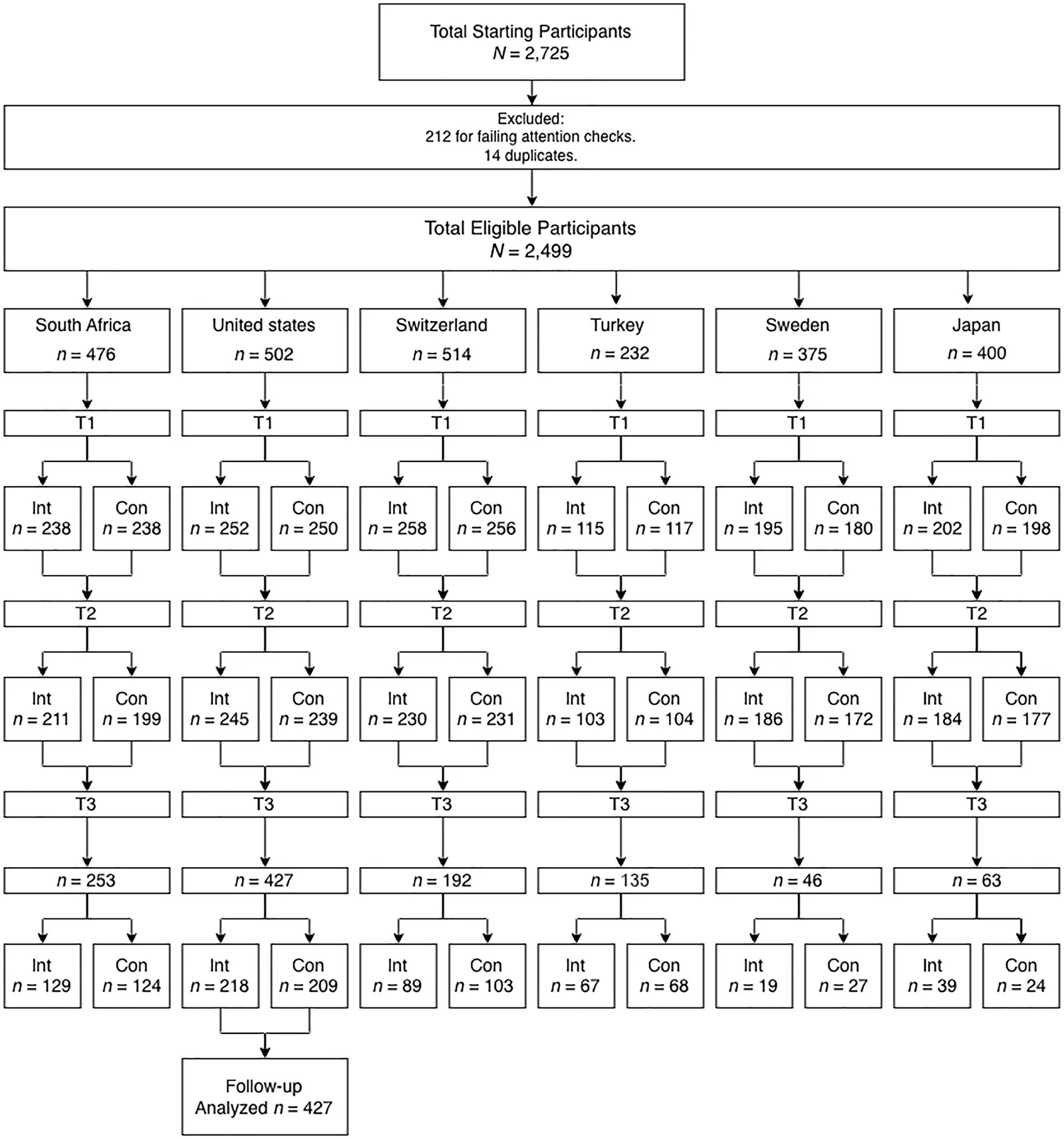
Follow-up (30-day post-randomisation; T3) analyses were not conducted for Japan and Sweden due to low response rates. Follow-up analyses were also not conducted for South Africa, Turkey, and Switzerland because no significant effects were observed at post-intervention (immediately post-randomisation; T2).

Between April 1st 2024 and August 29, 2025, 2725 participants were enrolled across six countries. Of these, 226 participants were excluded due to failed attention checks (n = 212) or duplicate responses (n = 14), resulting in a final analytic sample of 2499 participants. The total sample consisted of 400 from Japan, 476 from South Africa, 375 from Sweden, 514 from Switzerland, 232 from Türkiye, and 502 from the USA. Out of the analytic sample 1258 were allocated to the social contact-based intervention group and 1241 participants were assigned to the psychoeducational control group. Data collection began April 1st, 2024, and ended on August 29, 2025. The CONSORT flow of participants through the study is shown in Fig. 1.

Sociodemographic data for all countries are presented in Table 2. Age, gender, and education were not included as covariates in the linear mixed models as there was no empirical indication of baseline imbalance between groups.

**Table 2:** Sociodemographic baseline data for the total sample.

Variable	Psychoeducational control (*n* = 1241)	Social contact intervention (*n* = 1258)
Gender		
Male	456 (38.4%)	460 (37.7%)
Female	730 (61.5%)	758 (62.2%)
Age	26.94 + 5.17	27.09 + 5.25
Education		
Did not complete high school	28 (2.8%)	36 (3.6%)
High school graduate	580 (58.5%)	535 (53.3%)
Bachelor's degree	275 (27.7%)	307 (30.6%)
Master's degree or above	108 (10.9%)	125 (12.5%)
Subjective socioeconomic status	5.27 + 2.01	5.39 + 1.99
Prior treatment		
No	530 (43.2%)	569 (45.7%)
Yes, in the past	497 (40.5%)	488 (39.2%)
Yes, currently	200 (16.3%)	187 (15.0%)
CM scores		
Physical abuse	772 (62.2%)	759 (60.3%)
Emotional abuse	848 (68.3%)	855 (68.6%)
Sexual abuse	340 (27.4%)	338 (27.6%)
Emotional neglect	713 (57.4%)	696 (55.3%)
Physical neglect	252 (20.3%)	250 (20.7%)
Parental mental illness	467 (38.3%)	480 (38.1%)
Incarceration	168 (13.5%)	156 (12.4%)
Study Outcomes		
Perceived Recovery	9.09 (2.50)	9.10 (2.59)
Alienation	8.83 (3.06)	8.86 (3.02)
Stereotype Endorsement	6.27 (2.38)	6.25 (2.43)
Secrecy	5.90 (1.96)	5.85 (1.93)
Help-Seeking Intentions	8.99 (2.33)	8.87 (2.34)
Emotional engagement	8.59 (2.42)	8.83 (2.46)

Percentages reflect valid responses only; participants with missing data on a given variable were excluded from that variable's denominator.

Table 3 presents descriptive statistics and linear mixed-effects model (LME) results for all outcomes by condition. For secondary outcomes, p values were corrected for multiple comparisons using the Benjamini–Hochberg false discovery rate (BH-FDR) procedure (*k* = 4; *α* = 0.05).

**Table 3:** Descriptive statistics and adjusted between-group differences at post-intervention (linear mixed-effects models).

Outcome	Psychoeducational control (n = 1241)		Social contact intervention (n = 1258)		Adjusted between-group difference at post-intervention			
	Pre *M (SD)*	Post *M (SD)*	Pre *M (SD)*	Post *M (SD)*	Adjusted Mean Difference(AMD)	SE	95% CI	p
Focal subscale analysis								
Perceived Recovery	8.94 (2.49)	9.08 (2.59)	8.99 (2.56)	9.32 (2.63)	**+0.201**	0.064	[0.076, 0.327]	**0.002**^a^
Other Outcomes								
Alienation	8.81 (2.95)	8.36 (3.01)	8.81 (2.89)	8.30 (2.95)	−0.062	0.072	[−0.204, 0.080]	0.40
Stereotype Endorsement	6.39 (2.39)	6.11 (2.43)	6.33 (2.40)	6.06 (2.52)	−0.003	0.063	[−0.126, 0.120]	0.96
Secrecy	5.96 (1.91)	5.04 (2.07)	5.89 (1.89)	4.99 (2.13)	−0.008	0.069	[−0.143, 0.127]	0.91
Help-Seeking Intentions	9.00 (2.32)	9.36 (2.27)	8.88 (2.34)	9.18 (2.37)	−0.089	0.060	[−0.207, 0.029]	0.14

Intention-to-treat analysis: all N = 2499 randomised participants included. AMD = Adjusted Mean Difference at post-intervention (social contact intervention minus psychoeducational control); a positive AMD indicates greater improvement in the intervention group at post. SE = standard error. 95% CI = confidence interval for AMD. p (BH) = Benjamini–Hochberg FDR-adjusted p-value for secondary outcomes (k = 4).

Bold indicates statistically significant p-values (*p* < 0.05).

a Indicates *p* < 0.01.

1122 (90.4%) of 1241 participants assigned to the control group and 1159 (92.1%) of 1258 assigned to the intervention group completed the focal outcome (Perceived Recovery) after intervention, at a median of 12.1 min (IQR 9.5–16.1) and 11.7 min (IQR 9.2–15.9) after randomisation. At 30-day follow-up (US subsample only), 190 (76.0%) and 204 (81.0%) completed the focal outcome in the control and intervention groups respectively, at a median of 30.0 days (IQR 29.8–30.7) and 30.0 days (IQR 29.9–30.8) after randomisation.

Item-level missing data on outcome variables ranged from 7/2499 (0.3%; Help-Seeking Intentions) to 267/2499 (10.7%; Alienation). After intervention, missingness ranged from 27/2499 (1.1%; Help-Seeking) to 227/2499 (9.1%; Alienation). The number of participants with observed data at both before and after intervention under complete-case analysis ranged from 2137 (Alienation) to 2472 (Help-Seeking Intentions), representing 85.5%–98.9% of the randomised sample.

Missingness was unrelated to treatment allocation for all outcomes (all χ^2^ p > 0.14), supporting the missing at random (MAR) assumption. To satisfy the ITT requirement of including all randomised participants in every analysis, missing before and after intervention outcome scores were imputed using Multiple Imputation by Chained Equations (MICE; mean 20 imputed datasets). No covariates were included in the models; covariate-adjusted sensitivity analyses produced identical estimates, confirming successful randomisation. Prior to analysis, distributional properties of all outcome variables were examined. Skewness ranged from −0.67 to +1.21 and kurtosis from −0.60 to +1.32, indicating approximately normal distributions for four of the five outcomes; Stereotype Endorsement showed a moderate positive skew (1.21). Collinearity was assessed via variance inflation factors (VIF); all predictors yielded VIF <1.04, indicating no collinearity concerns. The statistical analysis plan was drafted by DA and SHN in November 2023, prior to data access by the statistician, and reviewed by the senior biostatistician (YS) before he accessed the data file in March 2025. The primary and secondary outcomes and the 1:1 allocation ratio were pre-specified in the trial registration collection.

**Fig. 2:**
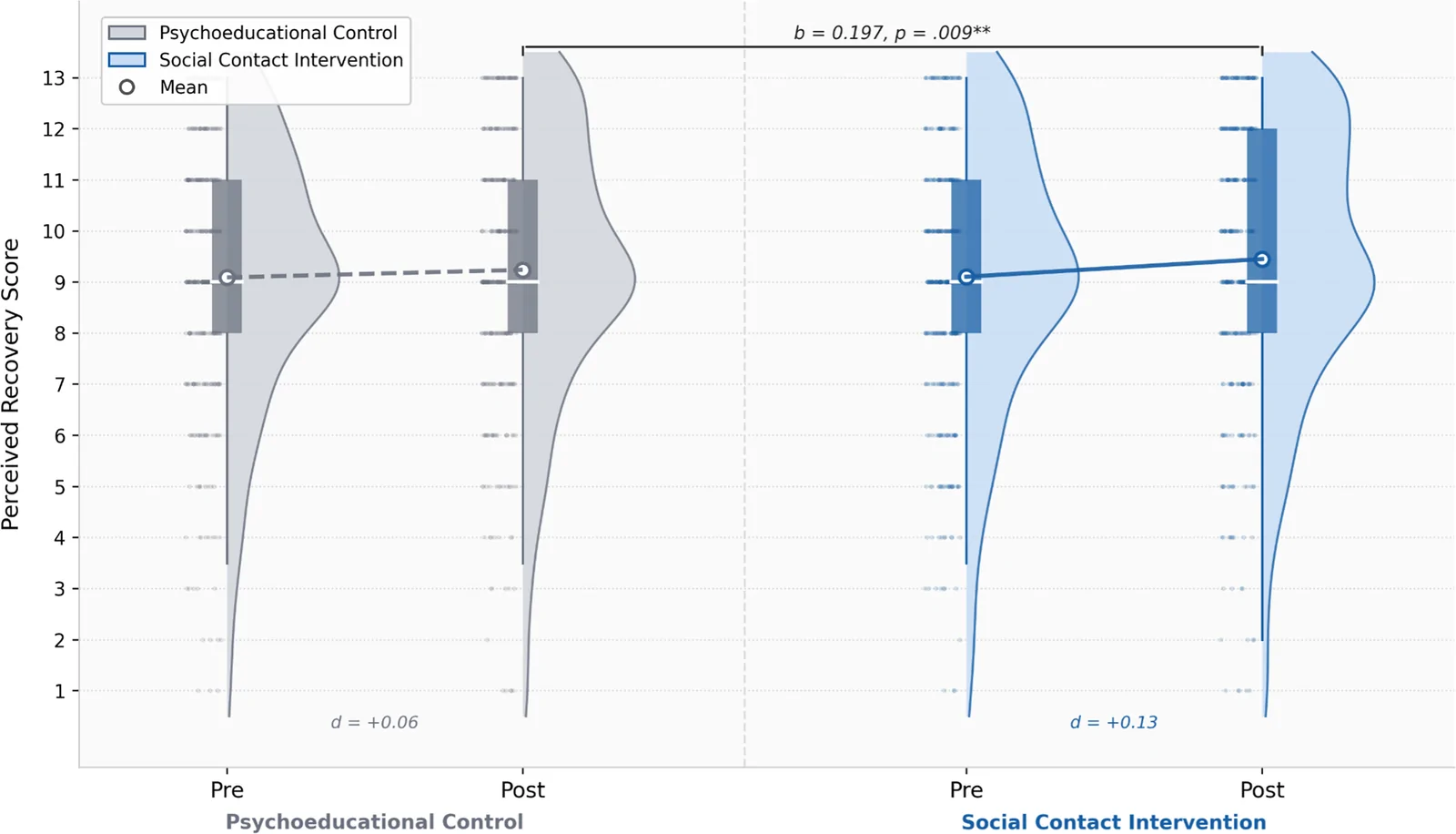
Perceived Recovery scores before and after the intervention in the psychoeducational control and social intervention groups. Violin plots show the distribution of scores, with mean values indicated by points. Within-group effect sizes (d) and the between-group effect (b) are shown.

At baseline, mean Perceived Recovery scores were comparable across conditions (control 8.94 [SD 2.49], intervention 8.99 [2.56]). Following the intervention, Mean recovery scores increased in both groups, with a larger gain in the social contact condition (after intervention 9.32 [2.63]) relative to the psychoeducational control (after intervention 9.08 [2.59]). After adjusting for pre-intervention scores, participants in the social contact intervention scored significantly higher on Perceived Recovery after intervention than those in the control condition (AMD 0.201, SE 0.064, 95% CI 0.076–0.327; p = 0.0020), indicating a small but statistically significant advantage in recovery-oriented self-beliefs (Fig. 2).

No significant adjusted between-group differences were observed for any other self-stigma domains and help-seeking intentions outcome after intervention, and all remained non-significant following BH-FDR correction.

After adjusting for baseline, Alienation scores did not differ significantly between conditions after intervention (AMD −0.062, SE 0.072, 95% CI −0.204 to 0.080; p = 0.40; p = 0.79).

Stereotype Endorsement declined modestly and comparably in both groups after intervention, with no significant adjusted between-group difference (AMD −0.003, SE 0.063, 95% CI −0.126 to 0.120; p = 0.96; p = 0.96).

For Secrecy, the adjusted between-group difference after intervention was negligible and non-significant (AMD −0.008, SE 0.069, 95% CI [−0.143 to 0.127]; p = 0.91; p = 0.96).

For Help-Seeking Intentions, there was no significant adjusted between-group difference after intervention (AMD −0.089, SE 0.060, 95% CI −0.207 to 0.029; p = 0.14; p = 0.55).

Due to the limited availability of data, a three-timepoint LME was estimated for Perceived Recovery among US participants. At 30-day follow-up, data were available for US participants only. At follow-up, adjusted Recovery scores (the estimated marginal mean) were comparable across conditions (control 9.32, intervention 9.37), and the adjusted between-group difference was non-significant (AMD 0.050, SE 0.181, 95% CI −0.306 to 0.407; p = 0.78), indicating no significant advantage of the social contact intervention over the psychoeducational control at 30 days.

Follow-up data collection was attempted across all six countries. In Sweden and Japan, follow-up data were largely unavailable due to technical difficulties with re-contacting participants via the recruitment platforms. In Türkiye, where recruitment relied on snowball sampling, follow-up contact was not feasible. In Switzerland and South Africa, sufficient follow-up data were collected; however, given the absence of significant pre–post-intervention effects in these countries, follow-up analyses were not pursued. Consequently, 30-day follow-up analyses were limited to the USA, where both sufficient data and a significant pre.

## Discussion

The present multi-country study examined the effectiveness of a brief, 2-min online social-contact intervention in reducing self-stigma and improving openness to treatment-seeking among young adults with a history of childhood maltreatment. The intervention produced a small but statistically significant improvement in participants’ sense of recovery and optimism relative to the psychoeducational control. However, this effect did not persist at 30-day follow-up among US participants, indicating that a single brief exposure is insufficient to produce durable change. This pattern is consistent with prior work demonstrating that single-dose brief interventions typically produce immediate but not lasting reductions in self-stigma.^26^^,^^37^ The value of the present findings therefore lies in demonstrating proof-of-concept—that a recovery-oriented narrative can produce an immediate shift in recovery beliefs—rather than in establishing standalone clinical effectiveness.

It is important to distinguish Perceived Recovery, as operationalised here using items from the Recovery Assessment Scale,^40^ from posttraumatic growth.^44^ Perceived Recovery reflects restored self-efficacy and hopefulness, a reduction in internalised helplessness, grounded in psychiatric rehabilitation theory. Posttraumatic growth, by contrast, refers to positive psychological transformation beyond the pre-trauma baseline (e.g., new possibilities, personal strength). These are related but conceptually distinct constructs. Perceived Recovery was retained as a focal subscale because it maps directly onto the intervention's narrative arc of hopefulness and agency. Contemporary recovery frameworks conceptualise personal recovery as involving processes such as hope and optimism about the future, identity, meaning, empowerment, and connectedness.^44^ No significant between-group differences were observed across the other self-stigma domains (Alienation, Stereotype Endorsement, Secrecy) or Help-Seeking Intentions. The selective improvement observed in Perceived Recovery may reflect the fact that the intervention narrative directly targeted recovery-oriented beliefs by depicting a survivor moving from shame and self-blame toward hope, agency, and recovery. This interpretation is consistent with contemporary recovery frameworks, which identify hope, identity, meaning, and empowerment as central components of personal recovery,^44^ and with the conceptualisation of recovery-oriented beliefs captured by the Recovery Assessment Scale^25^ This pattern is also consistent with prior evidence that shame- and concealment-based beliefs are more deeply rooted and less responsive to single brief exposures than recovery-oriented beliefs.^10^^,^^27^^,^^38^

Exploratory country-level analyses suggested cross-cultural variability in response to the intervention (Supplementary file 1), with country-specific participant characteristics presented in Supplementary Table S1 and exploratory country-level outcome analyses presented in Supplementary Table S2. These patterns should be interpreted as hypothesis-generating given the absence of pre-specified country-level hypotheses and the risk of inflated Type I error from multiple country-level comparisons.

Although brief, the intervention is highly scalable and can be delivered at virtually no cost across a range of settings that serve survivors of childhood maltreatment. Such videos could be incorporated into online screening platforms, primary care settings, trauma-focused services, community organisations, university counselling centres, and social media outreach campaigns. Because many survivors never reach speciality mental health care, brief recovery-oriented narratives may represent a practical first point of contact that normalises distress, conveys hope, and introduces the possibility of recovery. As digital mental health strategies continue to expand globally, brief social-contact interventions may serve as a feasible component of broader engagement and stigma-reduction efforts for trauma-exposed populations.

Several limitations should be acknowledged. First, although we used systematic forward–backward translation and video equivalence procedures, we did not conduct in-depth cultural adaptation in each country. It is therefore possible that some concepts (e.g., shame, disclosure) carried different connotations across languages, which could attenuate or distort intervention effects. Second, as noted, 30-day follow-up analyses were limited; in several countries, small sample sizes at follow-up precluded meaningful analysis. We therefore cannot speak to the durability of effects. Among the US subsample, the initial Recovery gain did not persist at 30-day follow-up, indicating that a single brief exposure was not sufficient to produce durable change. This is consistent with the broader stigma intervention literature, in which single-dose brief interventions typically produce immediate but not lasting reductions in self-stigma.^27^ Multi-dose or booster designs are likely needed to sustain initial gains. Third, outcomes relied on self-report and immediate post-exposure change; behavioural help-seeking was not captured beyond intention at this time point. Fourth, recruitment modalities differed by site (MTurk/CloudResearch vs. academic snowballing), which may introduce sampling heterogeneity. Fifth, multiple country-level tests increase the chance of Type I error; replication with prespecified country-level hypotheses is needed. Future work should examine whether co-designed, culturally adapted, and multi-dose versions of this approach produce more meaningful and durable change for childhood maltreatment survivors. Finally, whether individuals with very high baseline self-stigma respond differently to social contact narratives potentially experiencing upward social comparison rather than inspiration, whereby the protagonist's recovery is interpreted as pressure rather than possibility—remains an important question for future research that the present study was not powered to address.

Taken together, this multi-country randomised controlled trial demonstrates that a brief, 2-min social-contact video can generate small improvements in recovery-oriented self-beliefs among young adults with histories of childhood maltreatment across varied cultural contexts, but this effect did not persist at 30-day follow-up and did not extend to other self-stigma domains or help-seeking intentions. These findings support the proof-of-concept for recovery-focused narrative content as a potential entry point for digital stigma-reduction efforts, while underscoring that more intensive, multi-dose, or culturally adapted approaches are needed to produce meaningful and durable change in self-stigma among childhood maltreatment survivors.

## Contributors

SHN led the conceptualisation, intervention development, and trial design. DA, MO, SS, GA, BJ, US, and MP contributed substantially to the trial design. Trial implementation was conducted by SHN, DA, MO, SS, FA, CTF, DJ, JC, LY, SB, VS, GA, BJ, US, and MP across participating sites. SHN and YS had access and validated the study data. SHN led the data analysis with oversight from YS, AL, YN, and DA. SHN and YS accessed and verified the data. All authors contributed to the interpretation of the findings and to planning the manuscript. SHN wrote the first draft of the manuscript. All authors critically revised the manuscript and approved the final version. Study administration, protocol development, and implementation of the pilot study were led by SHN and CTF. Authorship was determined according to ICMJE criteria.

## Data sharing statement

De-identified data are available upon reasonable request to the corresponding author, pending approval from each participating site's institution. Data are not deposited in a public repository given restrictions imposed by site-specific ethics approvals and data-sharing agreements governing cross-national transfer of sensitive self-report data on childhood maltreatment and mental health. The statistical code used for the primary analyses is available from the corresponding author upon request.

## Declaration of interests

Julia Carranza-Neira recived Travel Support from NGO Zero to Three to attend Fellowship training on Infant and Early Childhood Mental Health. Stefanie Rita Balle presented the study's preliminary results at the 2025 DeGP Annual Meeting in Hamburg, but did not receive any honorarium or other compensation for doing so.

## Appendix A Supplementary data

Protocol
