# Supplementary material for: A brief video intervention targeting self-stigma among childhood maltreatment survivors: an international, multicentre, single-blind, randomised controlled trial
Source: eClinicalMedicine. 2026 Sep 15;100:104201. doi: 10.1016/j.eclinm.2026.104201 (PMC13594643; doi:10.1016/j.eclinm.2026.104201)
Supplement: Supplementary file 1 [file mmc1.pdf]

ClinicalTrials.gov

NIH National Library of Medicine
National Center for Biotechnology Information

Record 1 of 1

The U.S. government does not review or approve the safety and science of all studies listed on this website.

Read our full disclaimer (https://clinicaltrials.gov/about-site/disclaimer) for details.

Reducing Self-Stigma Among Individuals with History of Childhood Maltreatment: a Cross-Cultural Lens

ClinicalTrials.gov ID **NCT06159075**

Sponsor Research Foundation for Mental Hygiene, Inc.

Information provided by Yuval Y Neria, Research Foundation for Mental Hygiene, Inc. (Responsible Party)

Last Update Posted 2025-02-13

Completed

No longer looking for participants

Study Details Tab

Study Overview

Brief Summary

Individuals who report experiencing any kind of abuse during childhood report shame and self-blame, often leading to self-stigma and a reluctance to reveal their experiences and seek help. Such stigma may aggravate the mental health consequences of child maltreatment (CM).

The investigators hypothesize that:

1. The brief video-based intervention will have the immediate and repeated effect of reducing self-stigma among CM survivors compared with the control condition.
2. The brief video-based intervention will increase seeking treatment compared with the control condition.
3. The brief video-based intervention will show similar effects in reducing self-stigma across multiple countries.

Detailed Description

Prior research suggests that social contact-based interventions are the most efficient way of reducing stigma. This study aims to address self-stigma and empower individuals who self-reported experiencing any kind of abuse during childhood and increase their openness to seeking help, if needed.

Four hundred participants with self-reported experience of any kind of childhood abuse will be recruited to participate in a Randomized Controlled Trial (RCT) testing the efficacy of the intervention. Participants will be randomized into one of two arms: 1) A 2-minute video intervention in which a CM survivor (presented by an actor) shares their personal CM experience and describes how they were able to overcome feelings of shame and self-blame to seek mental health care; or 2) A 2-minute control video using the same actor, but without a personal narrative of CM experience. Both videos will be preceded by and immediately followed by questionnaires assessing self-stigma and openness to seeking treatment. An additional assessment of emotional engagement will be added immediately following intervention delivery. Following the intervention, there will be a 30- day follow-up to examine potential long-term effects.

The results will be analyzed alongside results from parallel protocols being conducted by partner sites in other countries (Switzerland, Sweden, Japan, Peru, India, Australia, Turkey, South Africa, and Israel). Each site will conduct the study under their own university's purview and with their own protocol, and only deidentified data will be shared for analysis.

All study procedures will be conducted remotely via CloudReseach and Mechanical Turk, which is a crowdsourcing platform. The study survey will be hosted by Qualtrics.

Official Title

Reducing Self-Stigma Among Individuals with History of Childhood Maltreatment: a Cross-Cultural Lens

Conditions

Stigma, Social | Child Abuse | Child Neglect | Help-Seeking Behavior

Intervention/Treatment

- Behavioral: Video

Other Study ID Numbers

- 8453a

Study Start (Actual)

2024-01-01

Primary Completion (Actual)

2024-04-01

Study Completion (Actual)

2024-04-01

Enrollment (Actual)

964

Study Type

Interventional

Phase

Not Applicable

Resource links provided by the National Library of Medicine

FDA Drug and Device Resources (https://clinicaltrials.gov/fda-links)

Contacts and Locations

This section provides contact details for people who can answer questions about joining this study, and information on where this study is taking place.

To learn more, please see the Contacts and Locations section in How to Read a Study Record (https://clinicaltrials.gov/study-basics/how-to-read-study-record#contacts-and-locations).

This study has 1 location

United States

New York Locations

New York, New York, United States, 10032
New York State Psychiatric Institute

Participation Criteria

Researchers look for people who fit a certain description, called eligibility criteria. Some examples of these criteria are a person's general health condition or prior treatments.

For general information about clinical research, read Learn About Studies (https://clinicaltrials.gov/study-basics/learn-about-studies).

Eligibility Criteria

Description

Inclusion Criteria:

- Age 18-80
- US resident
- Individuals who endorse yes to one of the following items: physical aggression in family or household, emotional or verbal abuse in family or household, sexual abuse or inappropriate sexual experiences, negligence - physical or emotional, mental illness or substance abuse at home, incarceration of family member
- Fluent in English and able to give informed consent

Exclusion Criteria:

- N/A

Ages Eligible for Study

18 Years to 80 Years (Adult, Older Adult)

Sexes Eligible for Study

All

Accepts Healthy Volunteers

Yes

Study Plan

This section provides details of the study plan, including how the study is designed and what the study is measuring.

How is the study designed?

Design Details

Primary Purpose : Other
Allocation : Randomized
Interventional Model : Parallel Assignment
Masking : Single (Participant)

Arms and Interventions

Participant Group/Arm	Intervention/Treatment
Experimental: Video with Childhood Maltreatment-Related Content<br><br>Participants will view a video of an actor describing the story of an individual who experienced childhood maltreatment and how they overcame its effects on their life.	Behavioral: Video<br><br>• A short video aimed at reducing self-stigma among individuals with a history of childhood maltreatment and increasing their help seeking intentions (if needed).
No Intervention: Control Video<br><br>Participants in this arm will view a same-length video with the same actor, but without a personal narrative of CM experience.	

What is the study measuring?

Primary Outcome Measures

Outcome Measure	Measure Description	Time Frame
Self-Stigma Questionnaire - Post-Intervention	Change in stigma items from baseline - higher score indicates higher stigma (range of 15 to 60)	Immediately post-intervention
Self-Stigma Questionnaire - Follow-Up	Change in stigma items from baseline - higher score indicates higher stigma (range of 15 to 60)	30 days post-intervention
Help Seeking Intentions - Post-Intervention	Change in treatment-seeking intentions from baseline - higher score indicates higher help-seeking intentions (range of 3 to 12)	Immediately post-intervention
Help Seeking Intentions - Follow-Up	Change in treatment-seeking intentions from baseline - higher score indicates higher help-seeking intentions (range of 3 to 12)	30 days post-intervention

Collaborators and Investigators

This is where you will find people and organizations involved with this study.

Sponsor

Research Foundation for Mental Hygiene, Inc.

Collaborators

- Columbia University

Investigators

- Principal Investigator: Yuval Neria, PhD, NYSPI and Columbia University

Study Record Dates

These dates track the progress of study record and summary results submissions to ClinicalTrials.gov. Study records and reported results are reviewed by the National Library of Medicine (NLM) to make sure they meet specific quality control standards before being posted on the public website.

Study Registration Dates

First Submitted

2023-11-28

First Submitted that Met QC Criteria

2023-11-28

First Posted

2023-12-06

Study Record Updates

Last Update Submitted that Met QC Criteria

2025-02-11

Last Update Posted

2025-02-13

Last Verified

2025-02

More Information

Terms related to this study

Additional Relevant MeSH Terms

Social Behavior
Behavior
Social Stigma
Help-Seeking Behavior
Tape Recording
Audiovisual Aids
Educational Technology
Technology
Technology, Industry, and Agriculture
Television
Videotape Recording

Plan for Individual Participant Data (IPD)

Plan to Share Individual Participant Data (IPD)?

No

Drug and device information, study documents, and helpful links

Studies a U.S. FDA-Regulated Drug Product

No

Studies a U.S. FDA-Regulated Device Product

No

Product Manufactured in and Exported from the U.S.

No
