# Supplementary material for: A brief video intervention targeting self-stigma among childhood maltreatment survivors: an international, multicentre, single-blind, randomised controlled trial
Source: eClinicalMedicine. 2026 Sep 15;100:104201. doi: 10.1016/j.eclinm.2026.104201 (PMC13594643; doi:10.1016/j.eclinm.2026.104201)
Supplement: Supplementary file 2 [file mmc2.docx]

**Supplementary file 1: Exploratory Analysis Per Country**

Exploratory country-level analyses revealed heterogeneity in intervention response. A significant Time × Group effect for Perceived Recovery emerged in the United States and Sweden, and a significant effect for Stereotype Endorsement emerged in Japan; no other country-level effects reached significance. These patterns should be interpreted as hypothesis-generating, given the absence of pre-specified country-level hypotheses and the elevated risk of Type I error from multiple comparisons.

Full demographic and outcome analyses appear in Supplementary Table 1 (sample characteristics) and Supplementary Table 2 (pre–post outcomes). Below is a brief overview of the main trends. Intervention and control groups were demographically comparable, with no significant differences in age, gender, or education (Supplementary Table 1). For the **USA**, only recovery showed a meaningful Group × Time effect, with the intervention producing a small but significant improvement, whereas the control group showed no change (Supplementary Table 2). For **Japan,** a significant interaction emerged only for stereotype endorsement: the intervention group showed a clear reduction, while the control group remained stable (Supplementary Table 2). No other outcomes differed by condition. For **Sweden**, the intervention produced a greater improvement in recovery compared to the control group (Supplementary Table 2).

**Participants**

Supplementary Table 1 presents the socio-demographic characteristics of the sample per country. Across the USA, Japan, and Sweden, participants in the control and intervention groups showed highly similar demographic profiles, with no significant differences in gender, age, or education (all p > .05). This indicates balanced group allocation and minimal risk of confounding.

**Supplementary Table S1: Socio-demographic characteristics of the sample of each country**

| **Variable** | **Psychoeducational Control** | **Social Contact Intervention** | **χ^2^** | **t** | **p** |
| --- | --- | --- | --- | --- | --- |
| **USA** |  |  |  |  |  |
| Gender |  |  | 0.49 |  | .51 |
| - Male | 105 (46.5%) | 102 (43.2%) |  |  |  |
| - Female | 121 (53.5%) | 134 (56.8%) |  |  |  |
| Age | 28.18±4.27 | 27.48±4.51 |  | 1.80 | .07 |
| Ethnicity |  |  |  | 3.88 | .42 |
| - African American | 40 (16.2%) | 49 (19.5%) |  |  |  |
| - Asian | 33 (13.4%) | 29 (11.6%) |  |  |  |
| - Native American | 3 (1.2%) | 7 (2.8%) |  |  |  |
| - White | 162 (65.6%) | 161 (64.1%) |  |  |  |
| - Other | 9 (3.6%) | 5 (2.0%) |  |  |  |
| Education |  |  | 3.09 |  | .37 |
| - I never completed high school | 0 | 0 |  |  |  |
| - High school graduate | 122 (48.8%) | 119 (47.2%) |  |  |  |
| - Bachelor’s degree | 96 (38.4%) | 96 (38.1%) |  |  |  |
| - Master’s degree or above | 32 (12.8%) | 34 (13.5%) |  |  |  |
| **Japan** |  |  |  |  |  |
| Age | 29.66±4.73 | 29.77±4.59 |  | 0.24 | .810 |
| Gender |  |  | 0.01 |  | .95 |
| - Male | 73 (37.6%) | 75 (37.9%) |  |  |  |
| - Female | 121 (62.4%) | 123 (62.1%) |  |  |  |
| Ethnicity |  |  |  | 4.76 | .19 |
| - African American | 0 (0.0%) | 1 (0.5%) |  |  |  |
| - Asian | 181 (92.3%) | 186 (92.5%) |  |  |  |
| - White | 14 (7.1%) | 9 (4.5%) |  |  |  |
| - Other | 1 (0.5%) | 5 (2.5%) |  |  |  |
| Education |  |  | 2.60 |  | .456 |
| - I never completed high school | 11 (5.6%) | 12 (6.0%) |  |  |  |
| - High school graduate | 107 (54.6%) | 98 (49.2%) |  |  |  |
| - Bachelor’s degree | 62 (31.6%) | 77 (38.7%) |  |  |  |
| - Master degree or above | 16 (8.2%) | 12 (6.0%) |  |  |  |
| **Sweden** |  |  |  |  |  |
| - Gender |  |  | 0.05 |  | .81 |
| - Male | 62 (34.6%) | 65 (33.5%) |  |  |  |
| Female | 117 (65.4%) | 129 (66.5%) |  |  |  |
| Age | 27.56±4.93 | 28.05±4.83 |  | 0.43 | .66 |
| Education |  |  | 1.21 |  | .75 |
| - I never completed high school | 15 (8.4%) | 15 (7.7%) |  |  |  |
| - High school graduate | 115 (64.2%) | 119 (61.0%) |  |  |  |
| - Bachelor’s degree | 28 (15.6%) | 39 (20.0%) |  |  |  |
| - Master degree or above | 21 (11.7%) | 22 (11.3%) |  |  |  |

**Pre-post effects by group**

Supplementary Table 1 presents the results of the countries in this study. Country-level LME models were estimated separately for each of the six participating countries across all five outcome variables. Only three models in three countries yielded statistically significant Time × Group interactions.

In the United States, the social contact intervention produced a significantly greater improvement in Perceived Recovery relative to the psychoeducational control at post-intervention (AMD = +0.325, SE = 0.123, 95% CI [0.083, 0.567], p = .009). The intervention group showed a larger pre-to-post gain (d = +0.18) than the control group (d = +0.07), consistent in direction and magnitude with the overall trial effect.

In Sweden, a significant adjusted between-group difference in Perceived Recovery was also observed at post-intervention (AMD = +0.425, SE = 0.184, 95% CI [0.064, 0.786], p = .021), representing the largest country-level Recovery effect across the trial. The intervention group demonstrated a meaningfully larger within-group improvement (d = +0.21) relative to the control group (d = +0.06).

In Japan, a significant adjusted between-group difference emerged for Stereotype Endorsement at post-intervention (AMD = -0.395, SE = 0.195, 95% CI [-0.779, -0.010], p = .044). The negative AMD indicates that the intervention group scored lower on self-stigmatising stereotype endorsement at post-intervention than the control group, after adjusting for baseline, with the intervention group showing a meaningful within-group reduction (d = -0.20) while the control group remained essentially unchanged (d = +0.03).

**Supplementary Table S2: Results of the countries in this study**

|  |  | **Psychoeducational Control** | | | **Social Contact Intervention** | | | **Adjusted Between-Group Difference at Post-Intervention** | | | |
| --- | --- | --- | --- | --- | --- | --- | --- | --- | --- | --- | --- |
| **Country** | **Outcome** | **Pre M (SD)** | **Post M (SD)** | ***d*** | **Pre M (SD)** | **Post M (SD)** | ***d*** | ***b*** | **SE** | **95% CI** | ***p*** |
| **United States** | Recovery | 9.39 (2.47) | 9.56 (2.50) | +0.07 | 9.65 (2.34) | 10.06 (2.35) | +0.18 | **+0.325** | 0.123 | [0.083, 0.567] | **.009 **** |
|  | Alienation | 8.89 (2.82) | 8.51 (3.00) | -0.13 | 8.77 (2.89) | 8.47 (3.01) | -0.10 | +0.097 | 0.168 | [-0.233, 0.427] | .563 |
|  | Stereotype | 6.37 (2.28) | 5.91 (2.30) | -0.20 | 6.22 (2.21) | 5.88 (2.38) | -0.15 | -0.039 | 0.112 | [-0.259, 0.180] | .724 |
|  | Secrecy | 5.81 (1.94) | 4.77 (2.09) | -0.52 | 5.85 (1.87) | 4.80 (2.05) | -0.53 | -0.177 | 0.143 | [-0.459, 0.105] | .218 |
|  | Help-Seeking | 9.27 (2.13) | 9.55 (2.12) | +0.13 | 9.28 (2.17) | 9.47 (2.24) | +0.09 | -0.087 | 0.117 | [-0.316, 0.143] | .459 |
| **Sweden** | Recovery | 8.97 (2.39) | 9.11 (2.44) | +0.06 | 9.11 (2.50) | 9.65 (2.60) | +0.21 | **+0.425** | 0.184 | [0.064, 0.786] | **.021 *** |
|  | Alienation | 8.77 (3.11) | 8.25 (3.11) | -0.17 | 8.74 (3.05) | 8.35 (3.14) | -0.13 | +0.096 | 0.209 | [-0.316, 0.508] | .647 |
|  | Stereotype | 6.27 (2.43) | 6.08 (2.52) | -0.08 | 6.07 (2.38) | 6.08 (2.46) | +0.00 | -0.097 | 0.149 | [-0.389, 0.196] | .516 |
|  | Secrecy | 5.97 (2.04) | 5.06 (2.13) | -0.44 | 5.89 (2.01) | 4.90 (2.15) | -0.48 | -0.176 | 0.147 | [-0.464, 0.112] | .231 |
|  | Help-Seeking | 8.82 (2.36) | 9.17 (2.21) | +0.15 | 8.94 (2.35) | 9.22 (2.25) | +0.12 | -0.067 | 0.149 | [-0.359, 0.226] | .654 |
| **Japan** | Recovery | 8.95 (2.57) | 9.13 (2.69) | +0.07 | 8.99 (2.77) | 9.19 (2.74) | +0.07 | +0.065 | 0.186 | [-0.300, 0.431] | .726 |
|  | Alienation | 8.80 (3.18) | 8.45 (3.19) | -0.11 | 8.83 (2.97) | 8.21 (3.10) | -0.21 | -0.042 | 0.207 | [-0.449, 0.365] | .839 |
|  | Stereotype | 6.12 (2.36) | 6.20 (2.55) | +0.03 | 6.47 (2.53) | 5.96 (2.67) | -0.20 | **-0.395** | 0.195 | [-0.779, -0.010] | **.044 *** |
|  | Secrecy | 5.62 (1.91) | 4.89 (2.07) | -0.37 | 5.75 (1.92) | 4.94 (2.25) | -0.40 | -0.016 | 0.167 | [-0.345, 0.313] | .925 |
|  | Help-Seeking | 9.13 (2.39) | 9.59 (2.18) | +0.20 | 8.90 (2.37) | 9.20 (2.35) | +0.13 | -0.211 | 0.140 | [-0.485, 0.064] | .132 |
| **South Africa** | Recovery | 9.32 (2.38) | 9.32 (2.62) | +0.00 | 9.26 (2.44) | 9.34 (2.66) | +0.03 | +0.098 | 0.188 | [-0.271, 0.467] | .602 |
|  | Alienation | 8.78 (3.04) | 8.17 (3.10) | -0.20 | 8.95 (2.98) | 8.60 (3.19) | -0.11 | -0.122 | 0.208 | [-0.532, 0.288] | .560 |
|  | Stereotype | 6.33 (2.35) | 5.73 (2.27) | -0.26 | 6.20 (2.40) | 5.82 (2.49) | -0.16 | +0.288 | 0.176 | [-0.058, 0.634] | .102 |
|  | Secrecy | 6.51 (1.60) | 4.71 (2.02) | -1.02 | 6.53 (1.61) | 4.88 (2.01) | -0.93 | +0.137 | 0.182 | [-0.222, 0.495] | .454 |
|  | Help-Seeking | 8.79 (2.46) | 9.11 (2.45) | +0.13 | 8.73 (2.45) | 8.87 (2.48) | +0.06 | -0.127 | 0.161 | [-0.443, 0.189] | .430 |
| **Switzerland** | Recovery | 9.07 (2.73) | 9.20 (2.78) | +0.05 | 9.11 (2.74) | 9.31 (2.75) | +0.07 | +0.097 | 0.171 | [-0.240, 0.433] | .573 |
|  | Alienation | 8.77 (3.17) | 8.43 (3.23) | -0.11 | 8.93 (3.14) | 8.51 (3.13) | -0.13 | -0.185 | 0.184 | [-0.547, 0.176] | .314 |
|  | Stereotype | 6.12 (2.38) | 5.92 (2.42) | -0.08 | 6.04 (2.41) | 6.10 (2.54) | +0.02 | +0.151 | 0.175 | [-0.193, 0.496] | .389 |
|  | Secrecy | 5.71 (1.92) | 4.89 (2.10) | -0.41 | 5.74 (1.91) | 4.94 (2.09) | -0.40 | +0.073 | 0.145 | [-0.212, 0.359] | .613 |
|  | Help-Seeking | 9.28 (2.27) | 9.50 (2.24) | +0.10 | 9.13 (2.20) | 9.37 (2.34) | +0.11 | -0.022 | 0.143 | [-0.302, 0.258] | .876 |
| **Türkiye** | Recovery | 8.66 (2.49) | 8.83 (2.82) | +0.07 | 8.96 (2.52) | 9.12 (2.78) | +0.06 | +0.041 | 0.219 | [-0.391, 0.473] | .851 |
|  | Alienation | 9.17 (3.06) | 8.38 (3.37) | -0.24 | 8.91 (3.14) | 8.49 (3.11) | -0.13 | -0.060 | 0.249 | [-0.551, 0.432] | .811 |
|  | Stereotype | 6.61 (2.44) | 5.97 (2.47) | -0.26 | 6.54 (2.50) | 6.00 (2.64) | -0.21 | +0.028 | 0.220 | [-0.406, 0.462] | .898 |
|  | Secrecy | 6.23 (1.27) | 4.68 (2.01) | -1.00 | 6.23 (1.27) | 4.66 (2.10) | -0.98 | -0.019 | 0.275 | [-0.562, 0.524] | .944 |
|  | Help-Seeking | 8.65 (2.44) | 9.36 (2.45) | +0.29 | 8.65 (2.54) | 9.25 (2.56) | +0.23 | +0.066 | 0.178 | [-0.284, 0.416] | .711 |

Note. AMD = Adjusted Mean Difference at post-intervention (social contact intervention minus psychoeducational control); Positive AMD = greater improvement in the intervention group. SE = standard error. d = Cohen’s d for within-group pre-to-post change (pooled SD). Complete-case analysis per country. Country-level analyses are exploratory and were not corrected for multiple comparisons; findings should be treated as hypothesis-generating. Shaded rows indicate AMD significant at p < .05.
